# Association between the home-to-healthcare center distance and hearing aid abandonment among older adults

**DOI:** 10.3389/fpubh.2024.1364000

**Published:** 2024-05-30

**Authors:** Eduardo Fuentes-López, Javier Galaz-Mella, Salvador Ayala, Carlos De la Fuente, Manuel Luna-Monsalve, Carrie Nieman, Anthony Marcotti

**Affiliations:** ^1^Departamento de Fonoaudiología, Escuela de Ciencias de la Salud, Facultad de Medicina, Pontificia Universidad Católica de Chile, Santiago, Chile; ^2^Faculty of Rehabilitation Sciences, School of Speech Therapy, Exercise and Rehabilitation Sciences Institute, Universidad Andres Bello, Santiago, Chile; ^3^Escuela de Odontología, Facultad de Odontología y Ciencias de la Rehabilitación, Universidad San Sebastián, Santiago, Chile; ^4^Exercise and Rehabilitation Sciences Institute, Postgraduate, Faculty of Rehabilitation Sciences, Universidad Andres Bello, Santiago, Chile; ^5^Escuela de Fonoaudiología, Facultad de Odontología y Ciencias de la Rehabilitación, Universidad San Sebastián, Santiago, Chile; ^6^Department of Otolaryngology-Head and Neck Surgery, Johns Hopkins School of Medicine, Baltimore, MD, United States; ^7^Cochlear Center for Hearing & Public Health, Johns Hopkins Bloomberg School of Public Health, Baltimore, MD, United States

**Keywords:** age-related hearing loss, hearing aids, geographic accessibility, follow-up care, hearing aid abandonment

## Abstract

**Background:**

Access to audiology services for older adults residing in sparsely populated regions is often limited compared to those in central urban areas. The geographic accessibility to follow-up care, particularly the influence of distance, may contribute to an increased risk of hearing aid abandonment.

**Objective:**

To assess the association between the home-to-healthcare-calibration-center distance and hearing aid abandonment among older adults fitted in the Chilean public health system.

**Methods:**

455 patients who received hearing aids from two public hospitals in two regions were considered. Univariate and multivariate Poisson regression models with robust variance estimation were used to analyze the association between the geographical distance and hearing aid abandonment, accounting for confounding effects.

**Results:**

Approximately 18% of the sample abandoned the hearing aid, and around 50% reported using the hearing aid every day. A twofold increase in distance between home and the hearing center yielded a 35% (RR = 1.35; 95% CI: 1.04–1.74; *p* = 0.022) increased risk of hearing aid abandonment. Also, those in the second quintile had a 2.17 times the risk of abandoning the hearing aid compared to the first quintile (up to 2.3 km). Under the assumption that patients reside within the first quintile of distance, a potential reduction of 45% in the incidence of hearing aid abandonment would be observed. The observed risk remained consistent across different statistical models to assess sensitivity.

**Conclusion:**

A higher distance between the residence and the healthcare center increases hearing aid abandonment risk. The association may be explained by barriers in purchasing supplies required to maintain the device (batteries, cleaning elements, potential repairs, or maintenance).

## Introduction

Several factors have been associated with hearing aid adherence, including sociodemographic variables such as income, education, and support from significant others (1, 2). Additionally, adherence is related to the technology and amplification provided by the device, patients’ self-perception of hearing loss, self-efficacy, and attitudes toward hearing aids (1, 2). Most available evidence considers adherence based on daily hearing aid use hours or days per week (3). On the other hand, complete abandonment of the device would expose individuals to the negative consequences of untreated hearing loss (4). Hearing aid abandonment has predominantly been studied in high-income countries, with its prevalence ranging from 1 to 57% (5). The perceived benefit and device competency, which may involve difficulties handling it, has been associated with abandonment (6). Costs of repairs and batteries, acoustic feedback problems (6), and negative attitudes toward (2) hearing loss and hearing aids have also been identified as reasons for abandonment.

Low- and middle-income countries (LMICs), such as Brazil (7), Colombia (8), and Chile (9), as well as high-income countries as Australia, Germany, and the United Kingdom, have implemented free public programs that provide hearing aids to older adults with hearing loss (10). Given the significant investment of public resources in this type of public policy and the potentially harmful effects of untreated hearing loss, it is essential to identify the variables associated with hearing aid abandonment in these programs. Several factors have been associated with the risk of hearing aid abandonment among older adults from the Chilean public health system (11). Income, self-perceived hearing loss, and satisfaction with the hearing aid were significantly associated with hearing aid abandonment, with the fifth quintile (highest income) being almost three times less likely to stop using their hearing aid than the first.

In Chile, since 2007, a public policy called the GES program (Garantías Explícitas en Salud in Spanish) has guaranteed that individuals aged 65 years and older with clinically significant hearing loss receive hearing aids at no cost or with a maximum co-payment of 20% of the device’s price. The co-payment amount is determined based on the individual’s income level (9). An otolaryngologist must prescribe the device based on pure-tone audiometric results, specifically a pure-tone average of ≥40 dB HL in the better ear. In this program, public hospitals provide hearing aids with multiple channels and signal processing programs at a low cost (approximately USD 220). In the GES program, one hearing aid is initially provided for 1 year. After that period, if the patient demonstrates adherence to the first hearing aid, a second hearing aid is provided in cases of bilateral hearing loss (binaural fitting) (9). Each hospital issues a tender for the contract to purchase hearing aids. Consequently, companies supply hearing aids and conduct follow-ups for fitted patients with limited territorial presence. It is typical for companies to have only one center for regions outside the country’s capital. Therefore, in implementing the GES program, which delivers hearing aids, additional sociodemographic variables such as geographical distance may be associated with device abandonment.

Geographic access to follow-up care, specifically the impact of distance to the center or hospital, has predominantly been studied in cochlear implant adult recipients. In the US, more than 80% of veterans in 7 states resided more than 180 miles from the nearest facility providing cochlear implant services (12). The geographic limitations for accessing cochlear implant care affect veterans living in rural and large urban population centers. Nassiri et al. found that among adults, there was an association between greater travel distances and older age at the time of cochlear implantation (13). Additionally, Davis et al. (14) observed a significant difference in socioeconomic position (SEP) between patients who attended and did not attend the evaluation appointment for cochlear implantation. Although travel time did not differ significantly between the two groups, there was a significant interaction between SEP and geographic location in North Carolina, where rural counties were farther from the cochlear implant center and were also more likely to be associated with lower SEP.

To the best of our knowledge, only one previous study conducted in Brazil reported that some patients missed follow-up appointments due to transportation difficulties (15). In their sample, a group of users relied on public transportation, taking multiple buses to attend appointments, which is financially burdensome and physically demanding, especially for older adults (15). However, it is essential to note that this information was self-reported, and the study did not specify the distance at which a significant effect on hearing aid abandonment would be observed.

It is crucial to recognize that disparities in geographic access to healthcare services are not limited to the traditional urban–rural division but can also be observed within suburban areas. This phenomenon is likely attributed to large cities’ rapid and continuous expansion, leading to the blurring of urban–rural boundaries. The availability and distribution of healthcare facilities can significantly influence the utilization patterns of health services, even within the urban radius of major cities (16). In Chile, outside the capital, hospitals serve large populations spread across different communes (the smallest administrative subdivision in Chile). Consequently, the hearing aid control centers associated with these hospitals may be located far from the homes of patients (i.e., 30 Km.), including those residing in suburban areas.

Given the potential influence of geographic factors, such as distance, this study aimed to assess the association between the home-to-healthcare-calibration-center distance and hearing aid abandonment among older adults fitted in the Chilean public health system. In addition to location, income and access to transportation play significant roles in shaping healthcare utilization patterns within large cities (17). On the other hand, social support from significant others is a crucial variable associated with hearing aid use (18). Since older adults have limited social security benefits in Chile (i.e., low pensions), it is possible to hypothesize that social support may modify the effect of geographical distance on the risk of hearing aid abandonment. A patient’s social network, either formal or informal, could provide financial support and facilitate access to transportation to the hearing aid control center.

According to the information above, we hypothesize that the distance from home to the healthcare calibration center may predict hearing aid abandonment among older adults in the Chilean public health system. Additionally, the effect of the distance from home to the healthcare calibration center on hearing aid abandonment may be modified by social support. Since the GES program has uniform requirements nationwide, we have established a cohort of older adult program beneficiaries living in two regions of Chile to test these hypotheses. To the best of our knowledge, this is the first study to specify the distance at which a significant effect on hearing aid abandonment was observed and their effect modifications.

## Methods

### Study design

This retrospective cohort study included older adults aged 65–85 who received hearing aids through the Chilean public health system. The study included patients from two hospitals in different regions of Chile, both serving similar populations of hearing aid beneficiaries due to the national uniform requirements of the GES program. However, interregional variations exist in the distribution of health services, particularly concerning the availability of otolaryngologists and the density of hearing health centers in urban and suburban populations within each region.

The study protocol was approved by the Scientific Ethics Committee of the Pontificia Universidad Católica de Chile, Santiago, Chile (ID: 221103002). Before the study began, all participants provided their informed consent by signing a consent form.

### Sample

Participants were recruited from La Florida hospital in the Metropolitan region, and Dr. Gustavo Fricke in Valparaíso region. We used a non-probabilistic sampling strategy, but randomly selected individuals who had received a hearing aid through the GES program. The hospitals maintained records of the individuals who had received hearing aids, and this information was obtained with prior authorization from an ethics committee. Participants selected were contacted by phone, informed about the study, and invited to participate. A team of 6 evaluators trained in administration of questionnaires visited participants at home.

### Sample size

The sample size for this study was determined based on an alpha level of 0.05, a power of 80%, and considering that the outcome was dichotomous. We also considered the hearing aid abandonment reported in a previous study among older Chilean adults by Fuentes-López et al. (11) When using regression models for a dichotomous outcome, it is recommended to include one predictor variable for every 10 events in the sample (19, 20). The study mentioned above reported that about 22% of the participants experienced hearing aid abandonment (event). Therefore, to include nine predictor variables in the analysis, we needed 90 events. By calculating 22% of 409, we determined that recruiting 409 participants would yield approximately 90 events (22% of 409 = 90 events). To account for a potential loss of 10%, we aimed to recruit 450 people for the study.

### Inclusion and exclusion criteria

Participants who self-identified as male or female, aged between 65 and 85 years, with bilateral sensorineural hearing loss greater than 40 dB (symmetrical or asymmetrical) as determined by audiometry, and who provided informed consent were included in the study. We excluded participants that exhibited any degree of cognitive impairment, scoring equal to or less than 12 points in the abbreviated version of the MMSE used in previous studies (11, 21). Additionally, individuals were excluded if they experienced communication difficulties unrelated to hearing problems (i.e., Aphasia) or had hearing pathologies unrelated to aging, such as middle or external ear abnormalities as based on an otoscopic examination.

### Variables and instruments

#### Hearing aid abandonment: primary outcome

Hearing aid abandonment was assessed through the question used in similar previous studies (11, 22): “Do you use your hearing aid?” The response options included: “Every day,” “Almost every day (at least five days a week),” “Some days (1–4 days a week),” “Almost never,” and “Never.” Additionally, for those who replied “never,” were questioned about the reasons for stopping use of their hearing aid, using a multiple-response question: “If you never use your hearing aid, please indicate the reason (all that apply).” The response options included: “No or little benefit,” “Situations with much noise bother me a lot,” “Low sound quality,” “Difficult to manipulate (control volume),” “Uncomfortable,” “Negative side effects (itching, rash, build-up of earwax),” and “Not necessary.” Participants were also given the option to specify other reasons for abandoning the use of hearing aids.

#### Geographic access: independent variable

Geographic access was determined based on the distance, measured in kilometers, from the users’ homes to the follow-up hearing care center. The addresses were collected from the participants and verified during the home visits. We obtained the follow-up hearing care center location from an electronic register of public providers (Mercado público in Spanish), which details the company awarded the bid for each hospital and time. Each company has a designated center for attending patients from a specific hospital. We used the geosphere package in the R software to calculate the minimum linear distance to the follow-up hearing care center using the “distHaversine” command. The “gmapsdistance” package in R was employed to access the programming interface of Google Maps for estimating the minimum travel distance using various transportation modes. This package, used in prior studies (23, 24), leverages Google Maps functions, enabling us to estimate the minimum travel distance for different means of transport, both public and private.

#### Social support: interaction variable

We evaluated social support through generic and specific instruments. We assessed the generic social support through the Medical Outcomes Study (MOS) questionnaire (25), which has been validated in Spanish (26). The MOS questionnaire is self-administered and comprises 20 items with a Likert-type format answers coded from 1 to 5 (from “None of the time” with 1 point to “All of the time” with 5 points). Furthermore, we assessed material-economic support using the question: “If you need any material assistance, companionship, or advice, do you have someone you can turn to?”

Instrumental and specific support received for using hearing aids was evaluated using The Glasgow Benefit Inventory (GBI) question (27): “Since getting your hearing aid, do you feel that you have had more or less support from your family?” (Possible answers ranged from “much more support” with 5 points to “much less support” with 1 point). Another support-specific question was formulated through the “Social Network Analysis” (28). This scale graphically represents the patient’s social networks, ranging from the closest and most intimate individuals to those less intimate but still significant. The patient was asked to indicate the number of people they would place within each level (outer, middle, inner). Further questions were posed: “Did any of the individuals depicted in the graph support maintaining or repairing the hearing aid, purchasing batteries, or learning to use the device?” Patients responded with a “Yes” or “No.” For those who responded positively, further inquiries were made, including “Who?” and “Which circle (level)?” in the social network graph.

#### Adjusting variables

#### Income

Income was evaluated through a question used in similar previous studies (11): In total, considering all your income, how much money do you usually receive per month? The person was told they should consider income from work (any kind of work, whether formal or not), help from family members in or outside the country, income from rental properties, income from a social security, or any other source of income.

#### Educational level

Years of formal education were obtained with the use of two questions (21). These questions were: (1) what is the highest educational level you have reached and (2) how many years did you attend school, including tertiary studies. Some participants did not directly recall the number of years they attended school, and thus, based on question 1, the number of years of formal education was obtained. In case the participants did not complete a certain educational level (preparatory, high school, or tertiary studies), they were assigned a number of years according to the last grade they reached.

#### Joint and visual problems

To assess joint and visual problems, we asked: “Has a doctor ever informed you that you have arthritis, osteoporosis, osteoarthritis, or any other joint problems?” The response options were “yes” or “no.” As for visual problems, participants were asked: “Without wearing glasses, how would you rate your eyesight for seeing things?” The response options included “Very good,” “Good,” “Fair,” “Bad,” and “Very bad.”

### Self-efficacy

The Spanish version of the “Measure of audiologic rehabilitation self-efficacy for hearing aids” (S-MARS-HA) questionnaire, validated by Fuentes-López et al. (21), was used to evaluate participants’ confidence in using and managing their hearing aids. Higher S-MARS-HA scores indicate more patient confidence in using and managing their hearing aids.

#### Attitudes toward hearing aids and hearing loss

To evaluate attitudes toward hearing aids and hearing loss, we utilized the Spanish version of the Hearing Attitudes in Rehabilitation Questionnaire (S-ALHQ) developed by Fuentes-López et al. (29). This questionnaire comprises 22 assertions that are organized into five subscales: *Denial of Hearing Loss*, *Negative Associations*, *Negative Coping Strategies*, *Manual Dexterity and Vision*, and *Hearing-Related Esteem*. Participants responded on a Likert-style scale, ranging from “strongly disagree” to “strongly agree.” Higher scores on each subscale indicate more negative attitudes toward hearing loss and hearing aids.

#### Self-perceived hearing and hearing thresholds

We assessed participants’ self-perceived hearing difficulties using a question from previous studies (11, 21). The question asked participants about their perception of hearing without using their device: “Do you believe you normally hear in both ears?” Also, the hearing thresholds were assessed according to the guidelines of the GES program. Audiometric testing using air conduction pure tone audiometry was conducted in a double-walled soundproof booth, covering frequencies from 0.25 to 8.0 kHz. The average air conduction pure tone thresholds (PTA) at 0.5, 1.0, 2.0, and 4.0 kHz were calculated for the ear with the hearing aid. The company responsible for fitting the hearing aid utilized this hearing assessment to calibrate the device.

#### Insertion gain

Using portable hearing aid analysis equipment (Interacoustics model Affinity Compact Version 4), the level of amplification provided by the hearing aid to the patient’s ear was obtained. This measure gave information about the hearing aid’s gain, inserted in the patient’s ear met the patient’s acoustic needs according to a prescriptive method (insertion gain). The difference between the patient’s needs and what the hearing aid provides was expressed in decibels (dB).

### Satisfaction with the device and improvement in quality of life

The satisfaction with the hearing aid device was evaluated using the International Outcome Inventory for Hearing Aids (IOI-HA) (30). Specifically, we analyzed the fourth question: ‘Considering everything, do you think your present hearing aid(s) is worth the trouble?’ Respondents provided Likert-type responses with five options ranging from ‘Not at all worth it’ to ‘Very much worth it.’ Additionally, to assess improvement in quality of life, we analyzed the seventh IOI-HA question: ‘Considering everything, how much has your present hearing aid(s) changed your enjoyment of life?’ The response options were in Likert-type format, ranging from ‘Worse’ to ‘Very much better.’

### Procedures

The hospital authorities provided a list of participants along with their contact information. We randomly selected participants from this list and contacted them via telephone to explain the study and invite them to participate. We used a script previously approved by an ethics committee to contact the patients. Those who agreed and consented to have their medical records reviewed were preselected for the study. The medical records were accessed to evaluate the presence of exclusion criteria, specifically external or middle-ear problems. Trained personnel visited the homes of the preselected participants to collect the data for the study. During the home visit, participants underwent a cognitive function assessment using a shortened version of the MMSE, with a maximum score of 19 points. Participants scoring 12 or below on the MMSE were excluded from the study. Those who met the inclusion criteria completed the questionnaires assessing the abovementioned variables.

Considering potential difficulties related to visual, the instruments were presented in printed form with visual support (Arial font size 40) to facilitate comprehension. Moreover, during the home interviews, the interviewer read the questions aloud to assist the participants further. It is worth noting that the total administration time for the instruments listed below was approximately 1 h and 45 min.

### Statistical analyses

The mean and standard deviation (SD) were reported for normally distributed continuous variables. Non-normally distributed variables were described using the median and percentiles 25 and 75th. Categorical variables were presented as relative and absolute frequencies.

Univariate Poisson regression models were used to analyze the association between the geographical distance and hearing aid abandonment. The selection of Poisson regression over logistic regression was driven by the prevalence of the outcome being greater than 10%. When the outcome is frequent, the odds ratio (OR) may exaggerate the effect size (31, 32). Risk ratios can be obtained by exponentiating coefficients from a generalized linear model with a log link and binomial outcome distribution (33). Poisson regression is a generalized linear model with a log link and a Poisson distribution (33). Thus, when employing Poisson regression with a binary outcome, the exponentiated coefficients represent risk ratios rather than incidence-rate ratios (33–36). Furthermore, applying a Poisson model in a study with a prevalent event could lead to wider 95% confidence intervals (95% CI) for the Relative Risk (RR), introducing bias into the estimate (37). However, estimating the adjusted RRs using a robust estimation method makes it possible to relax the assumption that the data follow a Poisson distribution without encountering the abovementioned estimation problem (33). Since the distribution of distances to the follow-up center exhibited a strong positive skewness (Supplementary Figure 1), a base-2 logarithmic transformation was used to enhance interpretation. Consequently, the RR indicates increased risk when the distance to the follow-up center doubles.

Subsequently, we constructed multivariate Poisson regression models to address confounding effects. To identify minimal covariates for adjustment, we utilized Directed Acyclic Graphs (DAGs), as described by Tennant et al. (38). DAGs offer a straightforward and transparent approach to illustrating prior knowledge, theories, and assumptions regarding variable relationships. By employing DAGs, we identified a set of adjustment variables that allowed for unbiased estimation of effects. The multivariate models were adjusted for: income, education, joint problems, visual acuity, perceived social and economic support, insertion gain, and geographical region. The number of follow-up appointments was included to block any potential mediated paths for estimating a direct effect. Additionally, independent predictors were incorporated to improve the model’s precision. To establish the robustness of the results, several sensitivity analyses were conducted. These involved constructing multivariate models with different combinations of adjusting variables and specifying multilevel Poisson regression models with two levels. In these models, the random intercept corresponded to the communes within the regions, encompassing a total of 9 communes.

## Results

### Sample description

The sample was composed of 455 patients who received hearing aids in the Metropolitan Region (62.86%) and in the Valparaíso Region. The median age of the patients was 79 (25^th^-75^th^: 75–83) years, with a slightly higher proportion of women (Table 1). The median years of education were 8 (25–75th: 5–12), with a median income of 260,000 Chilean pesos per month (25–75th: 185,000-400,000).

**Table 1:** Socio-demographic and clinical characteristics of participants (*n* = 455).

Characteristics	Median (Percentile 25–75th) or absolute frequency (%)
Age (in years)	79 (75–83)
Proportion of women	268 (59.03%)
Years of education	8 (5–12)
Monthly income (in Chilean pesos)	$260.000 ($185.000–$400.000)
Patients living within the Metropolitan region	286 (62.86%)
Pure-tone average (PTA in dB HL.)^a^	54 (46–63)
Self-reported joint problems	261 (59.80%)
Visual acuity self-report^b^	
Poor - very poor	137 (30.44%)
Fair	175 (38.89%)
Good - very good	138 (30.67%)
Self-reported hearing problems without hearing aid use^c^	432 (94.95%)

a Average of the audiometric thresholds at 0.5, 1.0, 2.0 and 4.0 kHz.

b Response to the question: “Without glasses, how is your vision for seeing things?”

c Response to the question: “Without using their device, do you believe you normally hear in both ears?” (Yes or No).

All patients demonstrated bilateral asymmetrical or symmetrical sensorineural hearing loss. The median pure-tone average was 54 dBHL (25–75th: 46–63). Additionally, around 95% reported hearing problems without the use of their hearing aid, and approximately 60% reported joint problems. A total of 23% of the sample reported bilateral hearing fitting. Further characteristics of the sample can be observed in Supplementary Tables 1, 2.

### Hearing aid abandonment

Approximately 18% (*n* = 81) of the sample abandoned the hearing aid (Table 2). The main reasons for hearing aid abandonment were discomfort in noisy situations (42.50%), no or limited benefit (26.25%), difficulties with manipulation (23.75%), and discomfort during use (25.0%). Additionally, around 50% reported using the hearing aid every day, and of those who used it, 50% reported using it throughout the entire day.

**Table 2:** Description of the hearing aid use, abandonment, and reasons for hearing aid abandonment (*n* = 455).

Variable	Proportion (%)
Weekly hearing aid use	
Every day	231 (50.88)
Almost every day (At least 5 days a week)	42 (9.25)
Some days (1–4 days a week)	62 (13.66)
Almost never	38 (8.37)
Never	81 (17.84)
Daily hearing aid use	
All day	211 (50.36)
A large part of the day	71 (16.95)
Half a day	33 (7.88)
Less than half a day	22 (5.25)
Only for short periods	82 (19.57)
Reasons for hearing aid abandonment (*n* = 81 non users)^a^	
No/poor benefit	21 (26.25)
Noisy situations are disturbing	34 (42.50)
Poor sound quality	15 (18.75)
Difficulties using it (for example, controlling the volume)	19 (23.75)
Poor fit and comfort	20 (25.00)
Negative side effects (for example, rashes, itching, pain, build-up of wax)	12 (15.00)
No need	10 (12.50)

a The patient could select more than one option.

### Distance/travel time between homes and follow-up hearing care center

Table 3 describes the distance/travel time between patients’ residences and follow-up centers. The median linear distance was 4.1 kilometers, 5.94 kilometers on public transportation, and 5.73 kilometers by car. Significant differences existed in the distances and travel time to the follow-up center between the two regions (Table 3).

**Table 3:** Distance/travel time between patients’ residences and follow-up hearing care centers (*n* = 455).

Variable	Total sample median (25–75th)	Metropolitan region median (25–75th)	Valparaiso region median (25–75th)	*p*-value for the difference^c^
Linear distance (km.)^a^	4.10 (2.63–5.57)	3.65 (2.32–4.78)	5.10 (3.53–11.63)	<0.001
Distance by public transportation (km.)^b^	5.94 (4.18–7.94)	5.33 (3.81–6.55)	9.18 (6.47–14.51)	<0.001
Distance by car (km.)^b^	5.73 (4.12–7.41)	5.06 (3.70–6.41)	8.12 (5.56–16.15)	<0.001
Travel time on public transportation (min)^b^	30.94 (23.01–37.21)	33.79 (28.53–38.5)	22.97 (19.27–29.92)	<0.001
Travel time on car (min)^b^	14.48 (10.66–17.53)	12.73 (9.62–5.67)	17.67 (14.52–23.58)	<0.001

a Minimum linear distance between patient residence and follow-up hearing care center using the geosphere package in R Studio software.

b Distance and travel time using three modes of transportation estimated using the GmapsDistance package in R Studio software and the Google Maps API.

c A non-parametric Mann–Whitney test was applied for assess the differences.

**Figure 1:**
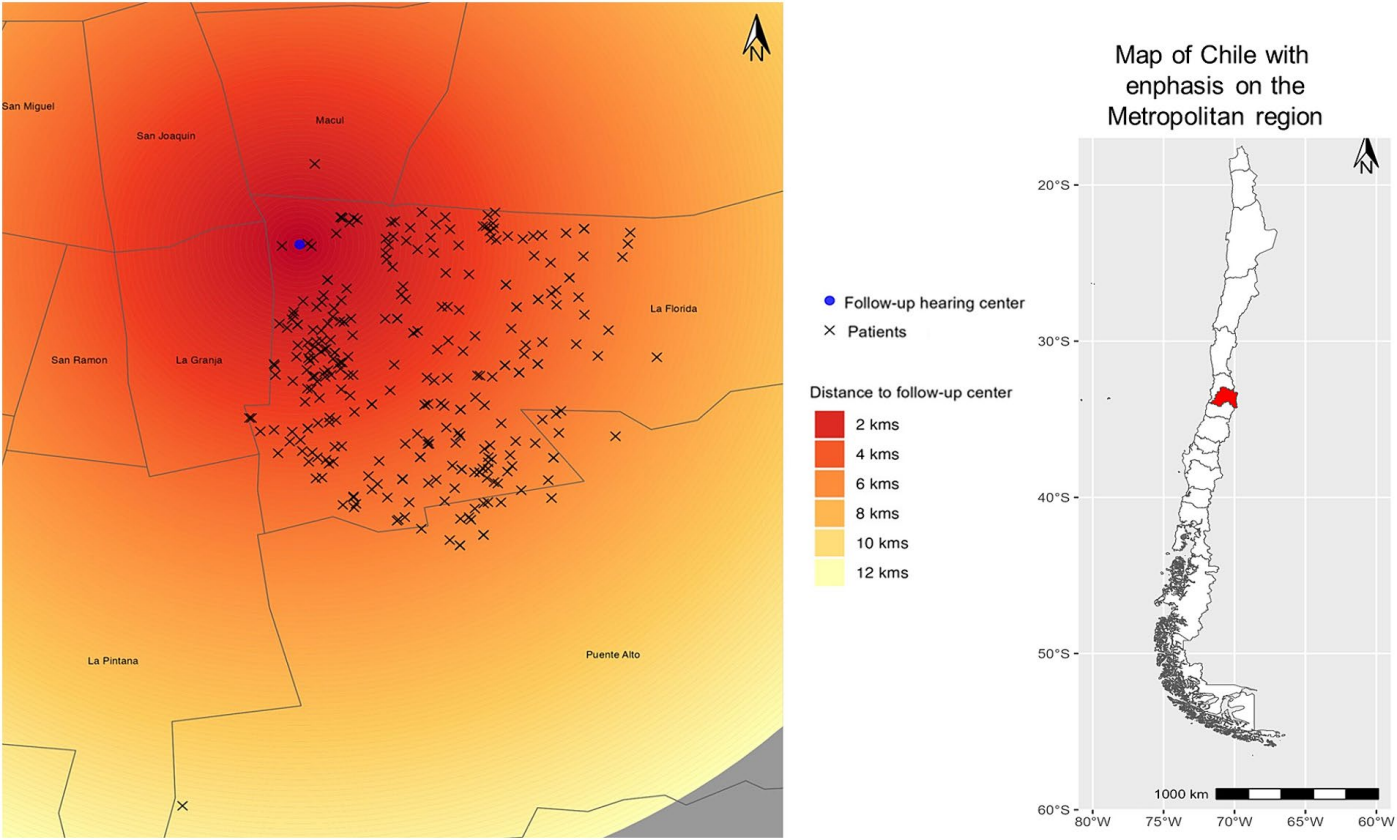
Distances from the patient’s homes to the follow-up healthcare centers in the Metropolitan region are described as heat map. Dark red shades indicate the shortest distances, while lighter shades of yellow represent great distances. The blue circle on the map represents the healthcare center’s location, and the “x” symbol denotes the patient’s home.

**Figure 2:**
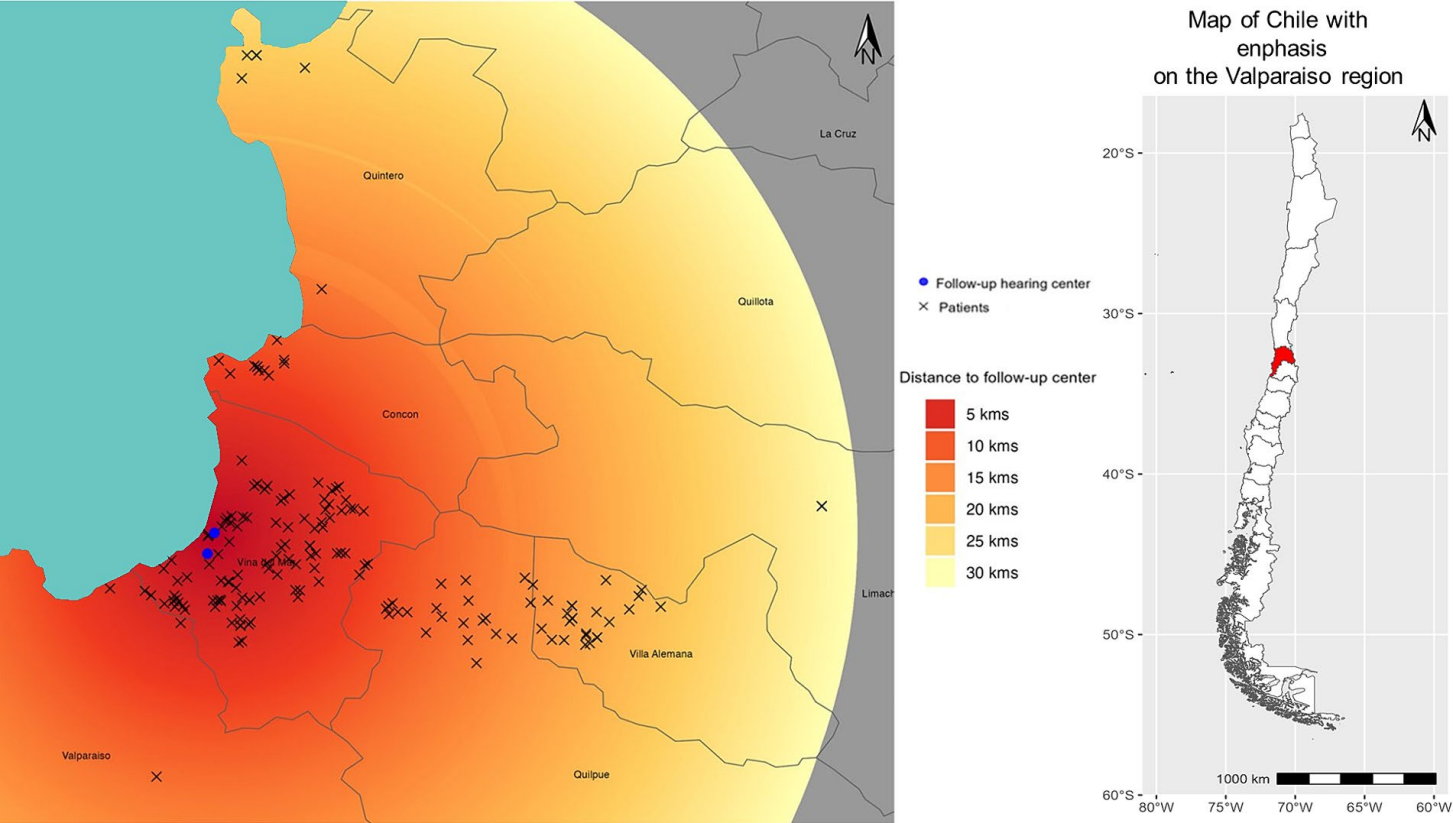
Distances from the patient’s homes to the follow-up healthcare centers in the Valparaiso region are described as heat map. Dark red shades indicate the shortest distances, while lighter shades of yellow represent great distances. The blue circle on the map represents the healthcare center’s location, and the “x” symbol denotes the patient’s home.

The locations of the patient’s homes and the follow-up centers in the two regions can be observed in Figures 1, 2.

### Variables associated with distance between homes and follow-up hearing care center

A positive and significant correlation was observed between education and distance to the follow-up hearing care center (rho = 0.11; *p* = 0.018). Additionally, significant positive correlations were found with pure-tone average (PTA; rho = 0.18; *p* < 0.001), number of follow-up appointments (rho = 0.16; *p* < 0.001), the difference between the target gain curve and amplification provided by the hearing aid (rho = 0.19; *p* < 0.001), and change in the quality of life with hearing aids (rho = 0.15; *p* = 0.001). Conversely, a significant negative correlation was found between the social support provided by the family for using the device and the distance to the follow-up hearing care center (rho = −0.10; *p* = 0.04), indicating that greater distance is associated with lower perceived familial support.

### Variables associated with the hearing aid abandonment in univariate analyses

When comparing those who abandoned their hearing aids and those who did not, significant differences were observed among participants who reported joint problems (RR = 1.71; *p* = 0.018), lower income (RR = 1.00; *p* = 0.047) and in attitudes toward hearing loss, specifically the denial factor (RR = 1.28; *p* = 0.042). The differences in the abovementioned variables represent an increased risk of hearing aid abandonment. On the contrary, generic social support (RR = 0.77; *p* = 0.010), the number of follow-up appointments (RR = 0.64; *p* = 0.025), self-efficacy (RR = 0.03; *p* < 0.001), satisfaction (RR = 0.47; *p* < 0.001) and improvement in quality of life with hearing aids (RR = 0.38; *p* < 0.001) exhibited a decreased risk of hearing aid abandonment.

The median distance to the follow-up center for those who abandoned the hearing aid was 4.21 km, while the median for those who continued using the hearing aid was 4.08 km. The unadjusted model showed no significant differences between groups (RR = 0.94; 95% CI: 0.78–1.12; *p* = 0.467).

### Association between geographical distance and hearing aid abandonment

Table 4 presents the results of the direct effect of geographical distance on hearing aid abandonment in three multivariate models with different combinations of adjustment variables. It is noteworthy that geographical distance was a significant predictor of abandonment when the model was adjusted for other predictors strongly associated with the outcome (as detailed in Table 4). In the second model, a twofold increase in distance corresponded to a 35% increased risk of abandonment (RR = 1.35; 95% CI: 1.04–1.74; *p* = 0.022). In the third model, only a social support variable was included to avoid collinearity, showing that a twofold rise in distance yields a 37% of abandonment risk (RR = 1.37, 95% CI: 1.06–1.76; *p* = 0.016).

**Table 4:** Multivariate Poisson regressions models to estimate the direct effect of geographical distance on hearing aid abandonment (*n* = 455).

Variable	RR (95% CI)^a^	*p*-value	RR (95% CI)^a^	*p*-value	RR (95% CI)^a^	*p*-value
Distance to the follow-up hearing care center (logarithmic transformed)^b^	1.02 (0.81–1.30)	0.844	**1.35 (1.04–1.74)**	**0.022**	**1.37 (1.06–1.76)**	**0.016**
Region (Metropolitan region as reference)	1.04 (0.90–1.18)	0.608	0.91 (0.77–1.09)	0.330	0.94 (0.80–1.12)	0.499
Years of education	1.01 (0.96–1.05)	0.627	0.99 (0.95–1.05)	0.692	0.99 (0.95–1.05)	0.964
Number of follow-up appointments attended	**0.63 (0.54–0.72)**	**<0.001**	**0.71 (0.59–0.83)**	**<0.001**	**0.70 (0.59–0.83)**	**<0.001**
Self-reported joint problems	**1.57 (1.01–2.45)**	**0.048**	**1.71 (1.01–2.89)**	**0.045**	1.65 (1.00–2.72)	0.050
Self-reported visual acuity (Poor-very poor as reference)^d^	0.90 (0.68–1.21)	0.514	0.89 (0.66–1.23)	0.503	0.84 (0.63–1.19)	0.235
General social support^e^	0.89 (0.72–1.11)	0.334	0.90 (0.71–1.14)	0.388		
General economic support^f^	1.01 (0.45–2.23)	0.986	1.22 (0.42–3.57)	0.717	1.76 (0.42–7.35)	0.437
Familiar social support with the device (Much more support as reference)^g^	0.81 (0.50–1.31)	0.405	0.71 (0.39–1.27)	0.254		
Presented economic support with the device^h^	0.81 (0.50–1.31)	0.398	1.05 (0.65–1.67)	0.852		
PTA (in dB HL.)	**0.98 (0.96–0.99)**	**0.024**	0.99 (0.96–1.01)	0.252	0.98 (0.96–1.01)	0.190
Income (logarithmic transformed)^c^	0.99 (0.70–1.39)	0.951	**1.69 (1.11–2.56)**	**0.013**	**1.90 (1.23–2.93)**	**0.004**
Difference with respect to the target gain			**1.06 (1.01–1.11)**	**0.023**	**1.07 (1.02–1.13)**	**0.006**
Self-efficacy^i^			**0.20 (0.05–0.87)**	**0.032**	**0.18 (0.05–0.65)**	**0.009**
Change in quality of life (Quality of life worsened)^j^			**0.48 (0.33–0.70)**	**<0.001**	**0.44 (0.30–0.63)**	**<0.001**
Attitudes toward hearing loss and hearing aids^k^			**1.34 (1.02–1.77)**	**0.035**	1.20 (0.88–1.62)	0.242
Satisfaction with the device (Very dissatisfied as reference)^l^					1.06 (0.79–1.43)	0.695
Self-reported hearing problems without hearing aid use^m^					0.44 (0.17–1.12)	0.086

Statistically significant results are highlighted in bold.

a Estimation obtained from a multivariate Poisson regression model with a logarithmic link and robust variance estimation.

b Geographical distance was transformed to a base-2 logarithm for better interpretation.

c Income was transformed to a base-2 logarithm for better interpretation.

d Response to the question: “Without wearing glasses, how would you rate your eyesight for seeing things?”

e Social support measured with the “MOS” social support questionnaire.

f Affirmative response to the question: “If you need any material assistance, companionship, or advice, do you have someone you can turn to?”

g Responses to the question from the “GBI” questionnaire: “Since getting your hearing aid, do you feel that you have had more or less support from your family?”

h Affirmative response to the question from the “Social Network Analysis”: “Did any of the individuals depicted in the graph support maintaining or repairing the hearing aid or ear mold, purchasing batteries, or learning to use the hearing aid?”

i Self-efficacy measured with the S-MARS-HA questionnaire.

j Responses to the question from the ‘IOI-HA’ questionnaire: Considering everything, how much has your present hearing aid(s) changed your enjoyment of life?”

k Responses to questions from the “Attitudes toward Hearing Loss Questionnaire (ALHQ)” Denial of Hearing Loss scale.

l Responses to the question from the ‘IOI-HA’ questionnaire: Considering everything, do you think your present hearing aid(s) is worth the trouble?

m Response to the question: “without using the device: “Do you believe you normally hear in both ears?”

### Sensitivity analysis

Three models similar to the previous table were created in order to perform a sensitivity analysis by incorporating other adjustment variables. In the first model of Table 5, satisfaction with the hearing aid, a variable strongly associated with the outcome, was included. In the second model, in addition to satisfaction, the degree of self-perceived hearing problem was incorporated, while in the third model, general social support was replaced with emotional support. In all sensitivity models, distance to the follow-up hearing care center emerged as a statistically significant predictor of abandonment. In the third model, for every twofold increment in distance, a 39% increase in risk of the hearing aid abandonment was observed (RR = 1.39, 95% CI: 1.07–1.82; *p* = 0.015).

**Table 5:** Sensitivity analysis for the effect of the geographical distance on hearing aid abandonment (*n* = 455).

Structure of the Poisson regression models with different combinations in the adjusting variables	RR (95% CI)^a^	*p*-value	RR (95% CI)^b^	*p*-value	RR (95% CI)^c^	*p*-value
Multivariate models without multilevel structure	**1.35 (1.04–1.74)**	**0.022**	**1.37 (1.05–1.80)**	**0.022**	**1.39 (1.07–1.82)**	**0.015**
Multivariate models with multilevel structure^d^	**1.35 (1.19–1.53)**	**<0.001**	**1.37 (1.21–1.55)**	**<0.001**	**1.39 (1.24–1.57)**	**<0.001**

Statistically significant results are highlighted in bold.

a Multivariate Poisson regression model adjusted for: income (logarithmic transformed), years of education, geographic region, self-reported joint problems, self-reported visual acuity, general social support, general economic support, familiar social support with the device, economic support with the device, PTA (in dB HL), number of follow-up appointments attended, difference with respect to the target gain, self-efficacy, change in quality of life, attitudes toward hearing loss and hearing aids (denial sub-scale), and satisfaction with the device.

b Multivariate Poisson regression model adjusted for the same variables as in the previous model, but adding self-reported hearing problems without hearing aid use.

c Multivariate Poisson regression model adjusted for the same variables as in the previous model, but changing general social support for emotional support.

d Multilevel Poisson regression models with two levels whose random intercept corresponded to the communes within the regions (9 communes).

Other sensitivity analyses can be observed in Supplementary Tables 3, 4. After dividing geographical distance into quintiles, we calculated the Population Attributable Fraction (PAF). Under the assumption that patients reside within the first quintile of distance (up to 2.3 km to the hearing center), a potential reduction of 45% (95% CI PAF: 12–65%; *p* = 0.013) in the hearing aid abandonment would be observed (Supplementary Table 3).

### Social support effect modification

Three multivariate models were constructed, incorporating interaction terms between geographic distance and generic and specific social support variables. The interaction between distance and emotional support, as evaluated by the MOS questionnaire, was non-significant (*p* = 0.938). Similar results were observed for the interactions with economic generic support (*p* = 0.237) and economic support with the device (*p* = 0.705).

## Discussion

The study aimed to assess the association between the home-to-healthcare-calibration-center distance and hearing aid abandonment among older adults fitted in the Chilean public health system. Hearing aid abandonment is multifactorial, in which the role of clinical variables and hearing aid-related technology has been investigated (2). Recently, the importance of broader social determinants of health in abandonment has been recognized (11, 39), as well the effect of sociodemographic variables on other individual/clinical predictors such as attitudes toward hearing loss and hearing aid use (29) and self-efficacy (21). In this present study, we included geographic access as another sociodemographic variable related to hearing aid abandonment. Our findings suggest that doubling the distance between a patient’s residence and the care center led to a 35% increase in the risk of hearing aid abandonment. The observed risk remained consistent across different statistical models.

The increased risk of hearing aid abandonment was associated with a greater distance to the follow-up hearing care center. Within the Chilean Public Health system, all device follow-up appointments are free and guaranteed by the GES program. Moreover, the statistical models allowed for estimating the direct effect, independent from attendance to the check-ups. This effect may be attributed to barriers in purchasing supplies necessary for maintaining the device’s normal functioning, such as batteries, cleaning elements, potential repairs, or maintenance. Individuals living farther from the follow-up hearing care center, which is located in the urban core, may rely on multiple modes of transportation to reach the follow-up center, thereby increasing time and travel-related costs. The Gustavo Fricke Hospital falls administratively under the Viña del Mar-Quillota Health Service, which is one of the largest in terms of geographical extension in Chile. For instance, residents of municipalities like Villa Alemana are, on average, 20–25 kilometers from the nearest hearing center.

There was considerable variability in the distance to the hearing care centers, with the 75th percentile being 4.8 km in the Metropolitan Region and 11 km in Valparaíso Region. This variability could be attributed to the greater number of municipalities associated with the Gustavo Ficke Hospital in the Valparaíso Region and its respective follow-up hearing care center. There are 18 municipalities where the Gustavo Ficke Hospital serves as a referral center for medical specialties, and these have a broader geographical distribution compared to the Metropolitan Region. Geographical distance has remained a longstanding barrier to healthcare within Chile. Scarpaci (40) observed that private healthcare was concentrated in areas with higher average household income among residents and in urban centers. While there has been an increase in the presence of primary care centers in Chile, the provision and follow-up related to hearing aids is still carried out by private companies with limited territorial presence (only one center for an entire region, in the case of Valparaíso).

The results are consistent with previous studies where geographical access affected the care for older adults using cochlear implants. Nassiri et al. (13) observed a correlation between travel distance and age of implantation. Also, older patients were more likely than younger patients to live in a rural residential area farther from the cochlear implant center. Davis et al. (14) in the United States found no relationship between distance and follow-up for patients with cochlear implants. However, they did find significant correlations with socioeconomic status, which also varied with distances to the care center. This result is similar to Cheung et al.’s study in Australia, where the authors found no relationship between distance and age of implantation among adults but did find a relationship with income and educational levels (41). The authors highlighted the potential compounding barriers of living in the last quartile of distance, far from urban centers, and having a low educational level. Differences between findings from the present study and existing literature could be attributed to previous studies not adjusting the effect of distance for other underlying variables, such as socioeconomic status. Furthermore, individuals navigating the healthcare system to obtain a cochlear implant may possess different sociodemographic characteristics than older adults who opt to receive free hearing aids.

Previously mentioned studies have included both urban and rural populations. In the present study, most of the sample came from urban-suburban areas. In the Valparaíso region, 8.4% of the population resides in rural areas; in the Metropolitan region, it is 3.1%. While residing in suburban areas is associated with better access to services compared to rural areas, the growth in healthcare supply has not been equitable. Specialized doctors in Chile’s public and private sectors are concentrated in urban centers, and follow-up centers for hearing aids are generally associated with a single hospital serving a large population.

Recent systematic reviews have shown that the degree of hearing loss severity is associated with hearing help-seeking and use of hearing aids (2, 42). Individuals with a greater degree of hearing loss are expected to have a greater intention to use their devices and be more willing to travel longer distances to a hearing care center. However, a greater degree of hearing loss might also be associated with requiring greater degrees of amplification from the device without necessarily leading to better speech recognition. This study found a correlation between greater distance and having a more severe degree of hearing loss, lower compliance with the required degree of amplification, and lower perceived support from close contacts related to the hearing aid. These could be patients with more severe hearing loss who need more adjustments of their hearing aids and might perceive that their environment does not provide sufficient support for the issues they face with their device.

Notably, the fact that individuals living in areas remote from urban centers exhibit specific characteristics may reflect barriers to obtaining hearing aids due to geographical distance. In Chile, there are a limited number of otolaryngologists outside of the capital city of Santiago, with places like Valparaíso having, for instance, half the number of otolaryngologists as Santiago (adjusted for population) (43). This unequal distribution is consistent with a study by Planey (44) in the United States, where audiologists were located in high-income areas, creating disparities in access, especially for older adults residing in rural regions.

Regarding the secondary objective, no significant interaction was observed between the effect of geographical access and the level of perceived social support on hearing aid abandonment. In essence, the impact of distance to the care center does not change with the degree of social support received. This result suggests that the challenge posed by geographical access extends beyond familial support. A study by Du et al. conducted in China found that many older adults seek company when traveling to healthcare centers, especially for longer distances (45). However, the location of healthcare facilities in urban areas may impose additional challenges on patients and their companions, including higher traffic congestion, increased travel expenses, and potential costs associated with work absenteeism.

### Limitations and projections

The findings of this study hold validity within the context of urban and suburban regions, potentially underestimating the effects in rural populations, where distances to healthcare centers could be considerably greater, along with the associated economic burden of travel. Moreover, the studied regions encompass the capital city and, in the case of Valparaíso, a neighboring region, which has superior access to services, public transportation, and specialists compared to more remote areas within Chile. A potential limitation is that several of the adjustment variables in the models could vary over time, such as attitudes and self-efficacy. Only a prospective longitudinal study could provide estimates free from potential reverse causality bias. Another limitation is that, although the sample consisted of new users who had used the device for at least 1 year, there may still be variability in the duration of hearing aid use (an unmeasured variable). The number of years of hearing aid use could be associated with the number of adjustments required for the device. Additionally, there may be an association between geographic distance and early abandonment of the device, with individuals living farther away from urban centers more likely to abandon their hearing aids prematurely. This aspect could be addressed in a future study. Furthermore, a limitation of having unbalanced groups in the distance variable is that, for the quartiles of greater distance, the smaller number of people can result in imprecise relative risk estimates (see Supplementary Table 3). Future studies should consider using more balanced quartile distance groups to address this limitation.

Indeed, it is important to highlight that the fieldwork for this study was conducted after lifting COVID-19-related quarantines, ensuring that individuals were unrestricted in their mobility. Additionally, abandonment rates in a preceding cohort study (11), with similar methodology, demonstrated a comparable percentage (18% [95% CI: 14.58–21.6] versus 21% [95% CI: 17.7–26.3]). Also, the uniformity of results across two distinct regions in Chile may be attributed to the standardized criteria for the distribution of hearing aids within the GES program. Notably, the technical features of the devices remain relatively consistent across regions, primarily falling within the mid-to-low range category.

One potential projection of the study could involve exploring alternative health centers beyond those of private companies for the management of patients’ follow-up needs. Considering the territorial presence and the greater availability of Primary Health Care (PHC) centers (46), it would be worthwhile to assess the feasibility of conducting hearing care follow-up in such centers. Crespo et al. (47) observed high accessibility and shorter distances to the primary care network in Chile in areas with lower income among residents. In our sensitivity analysis, assuming patients reside within the first quintile (up to 2.3 km.) of distance, a potential reduction of 45% in hearing aid abandonment could be observed. The median distance to the PHC from the patient’s home was 0.8 km in this population. Future studies should further evaluate the feasibility of implementing follow-up in PHC centers in Chile. Another projection is to employ a mixed-method approach, supplementing quantitative data with qualitative data. This approach could provide valuable insights into the autonomy of individuals when transporting themselves, helping us understand whether individuals travel independently or rely on family members for assistance. The mixed-method approach could also help us understand why individuals stopped attending the hearing-care center and discontinued using their hearing aids. Additionally, a follow-up study could shed light on the timing of both events.

## Conclusion

The study aimed to assess the effect of geographic access to follow-up care on hearing aid abandonment among older adults in the Chilean public health system. Our findings revealed that doubling the distance between the residence and the care center led to a 34% increase in the risk of hearing aid abandonment. The observed risk remained consistent across multiple statistical models. The effect might be attributed to barriers in purchasing supplies required to maintain hearing aids, such as batteries, cleaning elements, potential repairs, or maintenance. Since the median distance to the PHC from the patient’s home was 0.8 km, farther studies should evaluate the feasibility of implementing follow-up in the aforementioned centers in Chile.

## Data availability statement

The dataset used and analysed during the current study is available from the corresponding author on reasonable request.

## Ethics statement

The study protocol was approved by the Scientific Ethics Committee of the Pontificia Universidad Católica de Chile, Santiago, Chile (ID: 221103002). The study was conducted in accordance with the local legislation and institutional requirements. Before the study began, all participants provided their informed consent by signing a consent form.

## Author contributions

EF-L: Conceptualization, Data curation, Formal analysis, Funding acquisition, Investigation, Methodology, Project administration, Resources, Software, Supervision, Validation, Visualization, Writing – original draft, Writing – review & editing. JG-M: Conceptualization, Data curation, Formal analysis, Investigation, Software, Validation, Visualization, Writing – original draft, Writing – review & editing. AM: Data curation, Investigation, Project administration, Writing – original draft, Writing – review & editing. SA: Data curation, Formal analysis, Validation, Writing – original draft, Writing – review & editing. CF: Project administration, Supervision, Writing – original draft, Writing – review & editing. ML-M: Writing – original draft, Writing – review & editing. CN: Writing – original draft, Writing – review & editing.
