# Supplementary material for: Association between the home-to-healthcare center distance and hearing aid abandonment among older adults
Source: Front Public Health. 2024 May 30;12:1364000. doi: 10.3389/fpubh.2024.1364000 (PMC11169842; doi:10.3389/fpubh.2024.1364000)
Supplement: Supplementary file 1 [file Data_Sheet_1.docx]

**Supplementary material**

The distribution of distances to the follow-up center exhibited a strong positive skewness (Supplementary Figure 1).

**
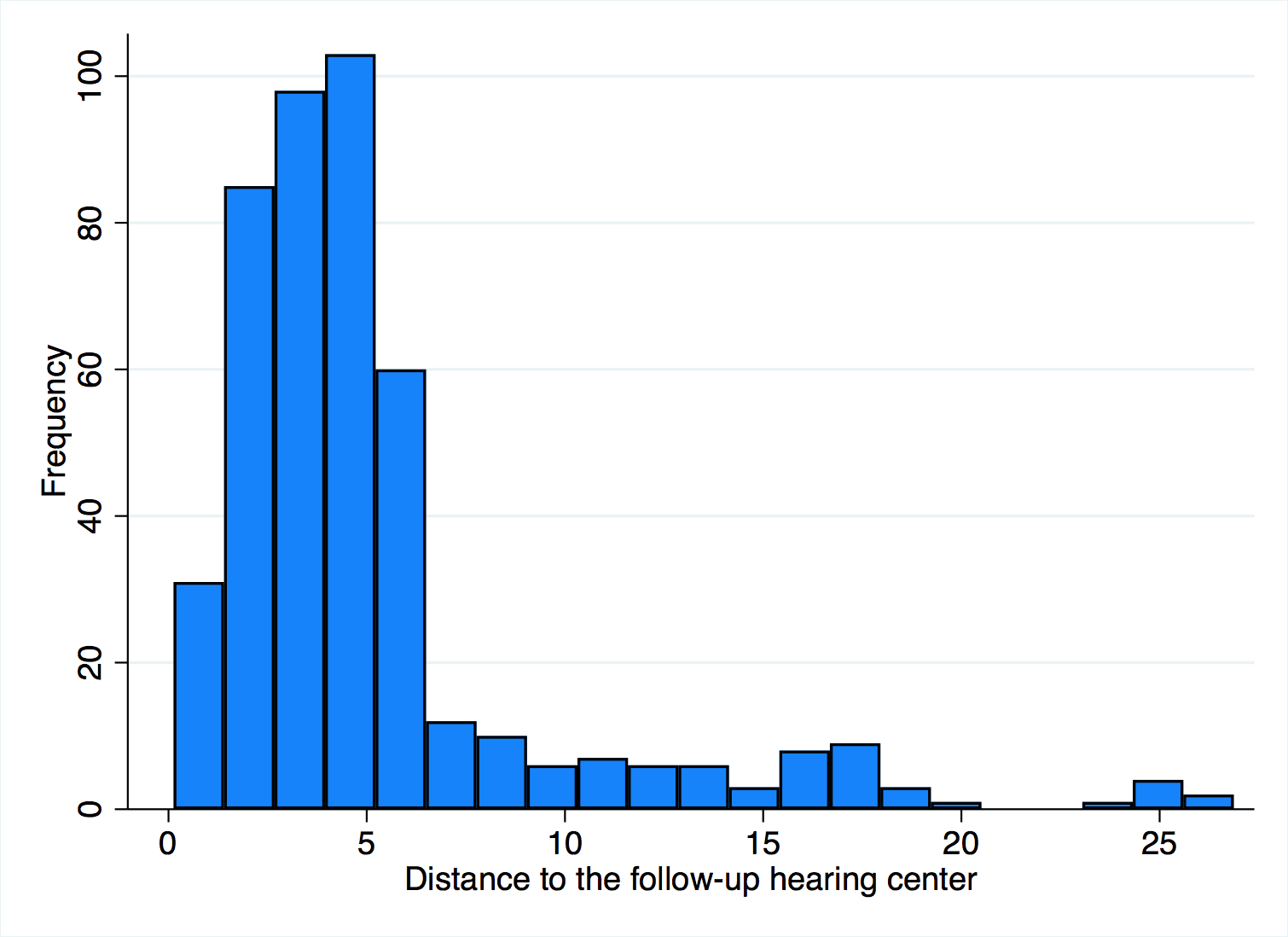
**

**Supplementary Figure 1.** Distribution of distances between patients home and the home-to-healthcare-calibration-center.

**Social support**

Approximately one-third of the patients reported experiencing positive changes in family support for hearing aid use after its delivery (see Supplementary Table 1). Regarding general social support measured by the MOS questionnaire, the median score was 5 points (25th-75th percentile: 4.36-5.00) on a scale of 1 to 5. Nearly 95% reported having someone who provided general economic support, and 30% indicated receiving assistance from someone in maintaining or repairing the hearing aid (specific support).

| **Supplementary table 1.** General and specific social support characteristics of the sample (n=455). | |
| --- | --- |
|  | **Median (Percentile 25^th^-75^th^) or frequency (%)** |
| MOS questionnaire score (General social support) | 5.00 (4.36-5.00) |
| If you need any material assistance, companionship, or advice, do you have someone you can turn to?" (General economic support)^a^ | 418 (94.14%) |
| Since getting your hearing aid, do you feel that you have had more or less support from your family? (Specific support)^b^ |  |
| Much more support *-* more support | 159 (35.20%) |
| *No change* | 291 (64.10%) |
| Much less support *- Less support* | 4 (0.88%) |
| Economic support for hearing aid use (Specific support)^c^ | 135 (30.13%) |
| ^a^ Affirmative response to the question: "If you need any material assistance, companionship, or advice, do you have someone you can turn to?"  ^b^ Responses to the question from the GBI questionnaire: " Since getting your hearing aid, do you feel that you have had more or less support from your family?" The response options were regrouped into three categories.  ^c^ Affirmative response to the question adapted from the Social Network Analysis questionnaire: "Did any of the individuals depicted in the graph support maintaining or repairing the hearing aid, purchasing batteries, or learning to use the device?" | |

**Clinical variables**

A median of three follow-up appointments' attendance was observed (Supplementary table 2). Concerning hearing aid functioning, the median difference between the amplification provided by the hearing aid and the target gain estimated through a prescriptive method was 4.5 dB (25^th^-75^th^: 1.8-8.5). The median score in the self-efficacy questionnaire was 72.5% (25^th^-75^th^: 60.4-80.4). Moreover, 72% of participants reported satisfaction with their hearing aids, rating them as "quite a lot worth it” or “very much worth it." Nearly 62% noted positive improvements in their quality of life through the use of hearing aids.

Attitudes toward hearing loss were evaluated using the ALHQ questionnaire and its respective subscales (ranging from 1 to 5). Notably, the median score for “Hearing-Related Esteem” was 2.00 (25^th^-75^th^: 1.00-3.00), and for the “Denial of hearing loss” scale, it was 2.33 (25^th^-75^th^: 1.66-2.83), indicating a higher degree of negative attitudes (Supplementary table 2).

| **Supplementary table 2.** Descriptive statistics of clinical variables of the sample (n=455). | |
| --- | --- |
| **Variables** | **Median (Percentile 25^th^-75^th^) or frequency (%)** |
| Number of follow-up appointments attendance | 3 (2-4) |
| Difference with respect to the target gain (in dB.) | 4.5 (1.8-8.5) |
| Self-efficacy (S-MARS-HA questionnaire global score)^a^ | 72.5 (60.4-80.4) |
| Satisfaction with the device^b^ |  |
| Not at all worth it – slightly worth it | 72 (16.44%) |
| Moderately worth it | 52 (11.87%) |
| Quite a lot worth it – Very much worth it | 314 (71.79%) |
| Change in quality of life^c^ |  |
| Worse – no change | 92 (20.91%) |
| Slightly better | 77 (17.50%) |
| Quite a lot better- Very much better | 271 (61.59%) |
| Denial of Hearing Loss subscale score^d^ | 2.33 (1.66-2.83) |
| Negative Associations subscale score^d^ | 1.00 (1.00-1.5) |
| Negative Coping Strategies subscale score^d^ | 3.42 (2.57-4.28) |
| Manual Dexterity and Vision subscale score^d^ | 2.00 (1.00-3.66) |
| Hearing-Related Esteem subscale score^d^ | 2.00 (1.00-3.00) |
| ^a^ Self-efficacy measured with the S-MARS-HA questionnaire.  ^b^ Responses to the question from the IOI-HA questionnaire: Considering everything, do you think your present hearing aid(s) is worth the trouble?. The response options were regrouped into three categories.  ^c^ Responses to the question from the IOI-HA questionnaire: 'Considering everything, how much has your present hearing aid(s) changed your enjoyment of life?'. The response options were regrouped into three categories.  ^d^ Attitudes toward hearing loss were evaluated using the ALHQ questionnaire and its respective subscales (ranging from 1 to 5). | |

**Additional sensitivity analyses and Population Attributable Fraction (PAF)**

Additional sensitivity analyses examining the relationship between home-to-healthcare-calibration-center distance and hearing aid abandonment among older adults are presented in Supplementary tables 3 and 4. The effect of geographical distance was consistent, indicating an elevated risk of hearing aid abandonment with increasing distance or travel time.

| **Supplementary table 3**. Sensitivity analysis of the association between the home-to-healthcare-calibration-center distance and hearing aid abandonment among older adults (n=455). | | | | | | |
| --- | --- | --- | --- | --- | --- | --- |
| **Variable** | **RR (95% CI) ^a^** | **P-value** | **RR (95% CI) ^b^** | **P-value** | **RR (95% CI) ^c^** | **P-value** |
| Distance to the follow-up center |  |  |  |  |  |  |
| First quintile | Reference | - | Reference | - | Reference | - |
| Second quintile | 1.59 (0.84-3.02) | 0.155 | **2.15 (1.08-4.27)** | **0.028** | **2.17 (1.13-4.18)** | **0.020** |
| Third quintile | 1.51 (0.80-2.85) | 0.209 | **2.18 (1.11-4.27)** | **0.023** | **2.09 (1.02-4.26)** | **0.044** |
| Fourth quintile | 1.75 (0.91-3.35) | 0.092 | 1.94 (0.89-4.24) | 0.097 | **2.14 (1.08-4.24)** | **0.030** |
| Fifth quintile | 1.25 (0.54-2.91) | 0.604 | **2.82 (1.22-6.49)** | **0.015** | **2.44 (1.10-5.40)** | **0.028** |
| Population Attributable Fraction (PAF) in the scenario where the population is located in the first quintile of distance (up to 2.4 km) | 0.28 (-0.11-0.54) | 0.138 | **0.45 (0.12-0.65)** | **0.013** | **0.44 (0.13-0.64)** | **0.010** |
| **Statistically significant results are highlighted in bold**  ^a^ Multivariate Poisson regression model adjusted for: income, years of education, region, self-reported joint problems, self-reported visual acuity, general social support, general economic support, familiar social support with the device, economic support with the device, PTA (in dB HL.), and number of follow-up appointments attended.  ^b^ Multivariate Poisson regression model adjusted for the same variables as in the previous model, but adding difference with respect to the target gain, self-efficacy, change in quality of life, and attitudes towards hearing loss and hearing aids (denial sub-scale).  ^c^ Multivariate Poisson regression model adjusted for: income, years of education, region, self-reported joint problems, self-reported visual acuity, economic general support, PTA (in dB HL.), number of follow-up appointments attended, difference with respect to the target gain, self-efficacy, change in quality of life, attitudes towards hearing loss and hearing aids (denial sub-scale), satisfaction with the device and self-reported hearing problems without hearing aid use. | | | | | | |

| **Supplementary table 4**. Sensitivity analysis of the association between the home-to-healthcare-calibration-center distance (Travel time on public transportation or car) and hearing aid abandonment among older adults (n=455). | | | | | | | | | | | |
| --- | --- | --- | --- | --- | --- | --- | --- | --- | --- | --- | --- |
| Travel time on public transportation | | | | | | Car travel time | | | | | |
| **RR**  **(95% CI)^a^** | **P-value** | **RR**  **(95% CI)^b^** | **P-value** | **RR**  **(95% CI)^c^** | **P-value** | **RR**  **(95% CI)^a^** | **P-value** | **RR**  **(95% CI)^b^** | **P-value** | **RR**  **(95% CI)^c^** | **P-value** |
| 0.89  (0.64-1.26) | 0.535 | 1.44  (0.95-2.19) | 0.088 | 1.44  (0.95-2.18) | 0.085 | 1.11  (0.75-1.64) | 0.601 | **1.57**  **(1.03-2.41)** | **0.038** | **1.63**  **(1.07-2.48)** | **0.024** |
| **Statistically significant results are highlighted in bold**  ^a^ Multivariate Poisson regression model adjusted for: income, years of education, region, self-reported of joint problems, self-reported visual acuity, general social support, economic general support, familiar support with the device, economic support with the device, PTA (in dB HL), and number of follow-up appointments attended.  ^b^ Multivariate Poisson regression model adjusted for the same variables as in the previous model, but adding difference with respect to the target gain, self-efficacy, change in quality of life, and attitudes towards hearing loss and hearing aids (denial sub-scale).  ^c^ Multivariate Poisson regression model adjusted for the same variables as in the previous model, but adding satisfaction with the device and self-reported hearing problems without hearing aid use. | | | | | | | | | | | |
